# Identification of bromodomain-containing proteins prognostic value and expression significance based on a genomic landscape analysis of ovarian serous cystadenocarcinoma

**DOI:** 10.3389/fonc.2022.1021558

**Published:** 2022-10-05

**Authors:** Juan Zhang, Yan Li, Ting-yu Fan, Dan Liu, Wen-da Zou, Hui Li, Yu-kun Li

**Affiliations:** ^1^ Department of Assisted Reproductive Centre, Zhuzhou Central Hospital, Xiangya Hospital Zhuzhou Central South University, Central South University, Zhuzhou, China; ^2^ Hunan Province Key Laboratory of Tumor Cellular and Molecular Pathology, Cancer Research Institute, University of South China, Hengyang, China

**Keywords:** ovarian serous cystadenocarcinoma, bioinformatic analysis, bromodomain-containing proteins, epigenetic modification, histone modification regulators

## Abstract

**Background:**

Ovarian serous cystadenocarcinoma (OSC), a common gynecologic tumor, is characterized by high mortality worldwide. Bromodomain (BRD)-containing proteins are a series of evolutionarily conserved proteins that bind to acetylated Lys residues of histones to regulate the transcription of multiple genes. The ectopic expression of BRDs is often observed in multiple cancer types, but the role of BRDs in OSC is still unclear.

**Methods:**

We performed the differential expression, GO enrichment, GSEA, immune infiltration, risk model, subtype classification, stemness feature, DNA alteration, and epigenetic modification analysis for these BRDs based on multiple public databases.

**Results:**

Most BRDs were dysregulated in OSC tissues compared to normal ovary tissues. These BRDs were positively correlated with each other in OSC patients. Gene alteration and epigenetic modification were significant for the dysregulation of BRDs in OSC patients. GO enrichment suggested that BRDs played key roles in histone acetylation, viral carcinogenesis, and transcription coactivator activity. Two molecular subtypes were classified by BRDs for OSC, which were significantly correlated with stemness features, m6A methylation, ferroptosis, drug sensitivity, and immune infiltration. The risk model constructed by LASSO regression with BRDs performed moderately well in prognostic predictions for OSC patients. Moreover, BRPF1 plays a significant role in these BRDs for the development and progression of OSC patients.

**Conclusion:**

BRDs are potential targets and biomarkers for OSC patients, especially BRPF1.

## Introduction

Ovarian serous cystadenocarcinoma (OSC) ranks eighth in terms of most commonly diagnosed and lethal mortality overall for gynecological oncology, resulting in all kinds of family problems and a huge social burden (1). Molecular medicine has devoted enormous resources to examining signaling pathways and the underlying molecular mechanisms of signalling (2, 3). In spite of this, the mortality rate for OSC is still high, and the five-year survival rate for women with advanced OSC is lower than 50% (2). Therefore, finding biomarkers and therapeutic targets that are effective in diagnosing and treating this malignancy is of great significance.

Bromodomains (BRDs), a group of evolutionarily conserved protein–protein interaction modules, can specifically recognize acetylated lysine (Kac)1,2 residues in histone tails and other substrates to epigenetically regulate gene transcription (4). As epigenetic readers, BRDs are also involved in gene fusions, resulting in the generation of diverse and frequent oncogenic proteins (4, 5). The sequence and structural similarities of BRD-containing proteins have led to their classification into eight subfamilies (5). According to a previous study, all BRDs have a distinct secondary structure with a left-handed four-helix bundle connected by two loops (ZA and BC) (6). The ZA and BC loops form a hydrophobic pocket, which coordinates acetylated lysine at the end of the histone tail (6). Tyr1125, Tyr1167, and Asn1168 are at the center of the acetyllysine binding pocket, and they serve as the most conserved residues (6). BRD subfamily I has four members, CECR2 (a chromatin remodeling factor) (7), BPTF (a transcription factor) (8), KAT2A (a Histone acetyl transferase) (9) and KAT2B (a Histone acetyl transferase) (9). Subfamily II includes 4 transcription factors, BRD2/3/4 (10–12) and BRDT (13), and a chromatin remodeling factor, BAZ1A (14). BAZ1B (15), BRWD3 (16), PHIP (17), BRWD1 (18), CREBBP (19), EP300 (19) and BRD8 (20) belong to subfamily III. The subfamily IV members includes 7 transcription factors, ATAD2 (21), ATAD2B (22), BRD1 (23), BRPF1 (24), BRPF3 (25), BRD7 (26) and BRD9 (27). Subfamily V includes SP140 (28), SP140L (29), SP100 (30), SP110 (31), TRIM24 (32), TRIM33 (33), TRIM66 (34), BAZ2A (35) and BAZ2B (7). Subfamily VI has a histone methyltransferase (MLL) (36) and a transcriptional regulator E3 SUMO ligase (TRIM28) (36). BRD subfamily VII has four transcriptional regulators, including TAF1 (37), TAF1L (38), ZMYND8 (39) and ZMYND11 (40). Subfamily VIII was including 3 chromatin remodeling factors, SMARCA2/4 (41, 42) and PB1 (43), and a methyltransferase, ASH1L (44). The dysregulation of these BRDs plays an important role in the development of inflammatory, autoimmune, and cancer diseases (45–48). However, the biological function and prognostic significance of these BRDs for OSC progression are still poorly understood.

**Figure 1:**
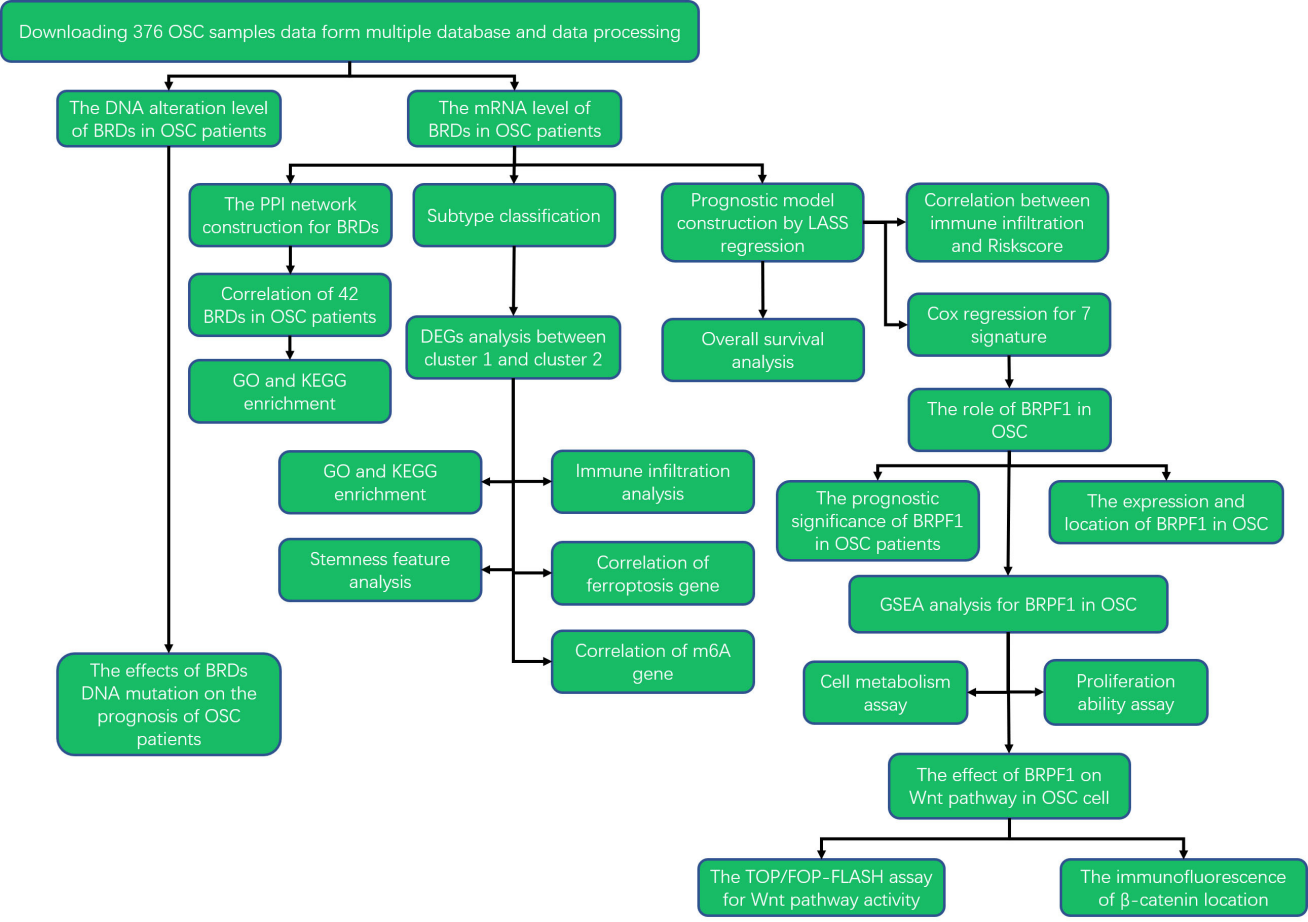
Flow chart of the study.

To elucidate the roles of these BRDs in the development and progression of OSC, we utilized The Cancer Genome Atlas (TCGA) database to confirm the mRNA levels of BRDs in OSC. Then, we also confirmed the DNA alteration level of BRDs by the cBioPortal database. Subsequently, we used the STRING database to construct a PPI network. Next, we predicted the biological functions and molecular pathways of BRDs in OSCs based on the DAVID database. Moreover, we performed subtype classification and LASSO modelling to further elucidate the functions and prognostic value of these BRDs in OSC progression based on the TCGA database. Finally, we found that BRPF1 was a significant BRG in the development of OSC. The BRPF1, as a subunit of the MOZ histone acetyltransferase (HAT), recognizes acetylated histones, such as H2AK5ac, H4K12ac, H3K14ac, H4K8ac, and H4K5ac (49). Moreover, several studies have reported that abnormal BRPF1 expression plays an important role in many cancer types, including liver cancer (50), medulloblastoma (51), and leukemia (52). But the role of BRPF1 in OSC progression is still unclear. Therefore, we further confirmed the effect of BRPF1 inhibition on OSC cell proliferation, glucose homeostasis and Wnt pathway activation. The research strategy is showed in **Figure 1**.

## Methods

### Bioinformatic expression analysis

We used the Cancer Genome Atlas (TCGA) (https://www.cancer.gov/tcga) database to confirm the mRNA levels of BRDs in **376** OSC patients (53), which contains clinical parameters, DNA alteration, and mRNA expression data for multiple cancer types. The Clinical Proteomic Tumor Analysis Consortium (CPTAC) database (https://proteomics.cancer.gov/programs/cptac) (54), an important protein expression database for many cancers, and the Human Protein Atlas (HPA) database (https://www.proteinatlas.org/) (55), an excellent protein expression database for cancer patients by IHC staining, were utilized to confirm BRPF1 protein expression and location in OSC patients. The Cancer Cell Line Encyclopedia (CCLE) database (https://sites.broadinstitute.org/ccle) was used to confirm the levels of BRPF1 in multiple OSC cancer cell lines. The database could prompt which cell lines was suitable for further cell experiment (56). The GTEX database (https://www.gtexportal.org/) was utilized to determine the mRNA levels of BRDs in 88 normal ovary samples (57). TIMER database (https://cistrome.shinyapps.io/timer/) was a systematical website for confirming the immune cell abundances by TIMER algorithm, including B cells, CD4+ T cells, CD8+ T cells, Macrophages, Neutrophils, and Dendritic cells which was used to confirm the correlation between immune infiltration and BRPF1 (58).

### DNA alteration analysis

The cBioPortal database (http://www.cbioportal.org/) was an open-access, open-source resource for elucidated cancer molecular profiles and clinical attributes, which was utilized to detect DNA alterations in BRDs in OSC patients (59).

### PPI network construction and GO enrichment

PPI network construction of 42 BRDs was based on the STRING database (https://cn.string-db.org/), which was an excellent website for constructing PPI network *via* utilizing proteomics and genomics data (60). The GO and KEGG enrichment analyses were based on the Database for Annotation, Visualization and Integrated Discovery (DAVID) database (https://david.ncifcrf.gov/) for these BRDs (61). LinkedOmics database (https://linkedomics.org/) was a nice website tool to confirm the expression, correlation, and gene functions of BRD-related genes in OSC patients, which was based on the TCGA database (62).

### Subtype classification

The “proportion of ambiguous clustering” (PAC) measure quantifies the middle segment. It is defined as the fraction of sample pairs with consensus indices falling in the interval (u1, u2) ∈ [0, 1], where u1 is a value close to 0 and u2 is a value close to 1 (for instance u1 = 0.1 and u2 = 0.9). A low value of PAC indicates a flat middle segment, and a low rate of discordant assignments across permuted clustering runs. We can infer the optimal number of clusters by the K value having the lowest PAC. Using the ConsensusClusterPlus package of R, 1000 iterations were used to evaluate cluster stability. Moreover, four-fifths of the total sample was drawn 100 times, clusterAlg = ‘hc,’ innerLinkage = ‘ward D2,’ Clustering heatmaps were generated using the ‘pheatmap’ package in R. A hierarchical clustering algorithm and heatmaps were drawn using the R package pheatmap v1.0.12. Heatmaps of gene expression were only used when SD > 0.1 was used. Then we used the GO and KEGG enrichment analysis to obtain the molecular functions between different clusters. Immune infiltration, stemness features, and drug sensitivity analysis were carried out to compare the molecular properties between two clusters. All statistics were performed using the R package.

### Prognostic model construction

Prognostic model can be used to study the prognosis of a series of genes on a tumor samples, based on the lasso or multivariable cox iterative regression method for dimension reduction and build a prognosis model (model contains the number of genes will not necessarily equal to input the number of genes) or directly through the multi-factors cox model building (model contains gene number is equal to input the number of genes, Note the number of input genes). The model is a RiskScore formula containing multiple genes. Each gene has a weight. Negative numbers represent the gene as a protective gene, while positive numbers represent the gene as a dangerous gene. The difference between Signature and nomogram prognostic models is that the former can only include genetic factors, while the latter can include clinical factors. After converting count data to TPM and normalizing the data log2 (TPM+1), normalized transcription data were used to calculate gene expression on the basis of log2 (TPM+1). In addition, missing and incomplete samples were deleted when clinical information was merged. Next, there were 376 OSC samples for subsequent analysis. The survival difference between groups was compared using the log-rank test. We evaluated the predictive accuracy of the risk score in all datasets using time-dependent ROC curve analysis (v 0.4). The LASSO regression algorithm was utilized for feature selection, 10-fold cross-validation was used, and the R package glmnet was used for the analysis. The R software ggstatsplot package was used to draw the correlations between gene expression and immune score. To visualize differentially expressed genes, the “pheatmap” package in R software was used. The RiskScore formula = Σ(the expression amount of each gene multiplied by the corresponding coefficient). According to the median risk score, we divided the patients into two groups. All statistics were performed using the R package. A p value <0.05 was considered statistically significant.

### Stemness and immune infiltration analysis

The detailed bioinformatic methods are described in our previous article (63).

### Cell culture and transfection

Human ovarian cancer cells (SKOV3) were purchased from the American Type Culture Collection (ATCC, VA, USA) and cultured in DMEM containing 10% (v/v) foetal bovine serum (FBS; Gibco, Invitrogen, Carlsbad, CA, USA) and 1% penicillin/streptomycin (GIBCO, CA, USA). All cells were incubated at 37°C and 5% CO (2). BRPF1 shRNA and empty vector plasmids were purchased from HonourGene (Changsha, China). For transient cell transfection, SKOV3 cells were seeded in 6-well plates and transfected with 3 μg empty vector and 3 μg BRPF1 shRNA plasmid using Lipofectamine 3000 (Invitrogen, Carlsbad, CA, USA) according to the protocol to establish a cell line with BRPF1 knockdown.

### Proliferation analysis

For MTT analysis, five thousand SKOV3 cells were seeded into 96-well plates for 24, 48, and 72 hours. In the subsequent step, the cells were treated with 0.5% MTT solution (5 mg/ml, Sigma−Aldrich; Merck KGaA) for 4 hours. Then, the MTT solution was removed, and DMSO was added. Cell numbers were calculated from an absorbance measurement at 490 nm. For EdU analysis, cell proliferation was evaluated with an EdU kit (RiboBio, Guangzhou, China). All assays were repeated three times.

### ATP, glucose, LD, and ROS measurements

An ATP assay kit (NJJCBIO, A095-1-1) was used to confirm the ATP level. A glucose kit (glucose oxidase method) (NJJCBIO, A154-1-1) was used to confirm the glucose level. A lactic acid assay (NJJCBIO, A019-2-1) kit was utilized to measure the lactic acid level. A reactive oxygen species assay kit (NJJCBIO, E004-1-1) was utilized to measure the ROS level. In these experiments, the OD values were measured at wavelengths of 505 nm (for glucose), 525 nm (for ROS), 530 nm (for lactic acid) and 636 nm (for ATP) after the reagents were mixed step by step according to the protocol.

### Immunofluorescence

Cells were fixed for 5 minutes in 100% methanol, followed by permeabilization for 5 minutes in PBS with 0.1% Triton X-100. Then, the cells were incubated in 10% normal goat serum for 1 h to block nonspecific protein−protein interactions. Then, the cells were incubated with the antibody anti-β-catenin (Abcam, ab32572, 1:250) overnight. Following primary antibody incubation, a secondary antibody (Abcam, ab150079 1 µg/ml) was used to label the cells. Two hours later, 0.1% DAPI was used to stain the nucleus for 15 min. Images were detected by confocal microscopy (Leica, Jena, Germany).

### Dual-luciferase reporter gene

M50 Super 8x TOPFlash (VT8105) plasmid and M51 Super 8X FOPFlash (TOPFlash Mutant; VT8196) plasmid were purchased from Ubo Bio. Vector plasmid and M50 Super 8X TOPFlash were cotransfected into SKOV3 cells at a transfection ratio of 10:1, which was used as the control group of TOPFlash. BRPF1 shRNA and M50 Super 8X TOPFlash were cotransfected into SKOV3 cells at a transfection ratio of 10:1, which were used as the BRPF1 knockdown group of TOPFlash. Vector plasmid and M51 Super 8X FOPFlash were cotransfected into SKOV3 cells at a transfection ratio of 10:1, which were used as the control group of FOPFlash. BRPF1 shRNA and M51 Super 8X FOPFlash were cotransfected into SKOV3 cells at a transfection ratio of 10:1, which were used as the BRPF1 knockdown group of FOPFlash. Each group had 3 auxiliary wells. After successful transfection, substrate was added, and luciferase activity was measured..

### Western blot

Please refer to our previous article for specific methods (64). In brief, the extracted proteins were collected, denatured, and electrophoresed through a 10% SDS-polyacrylamide gel. The samples were loaded, and electrophoresis was performed for 80 min followed by transfer to PVDF membranes and blocking in 5% skimmed milk at 37°C. After shaking for 2 h, the membranes were incubated with primary antibodies (BRPF1, Abcam, Cat. No. ab282024, 1:500 dilution; beta-actin, Abcam, Cat. No. ab6276, 1:1,000 dilution) at 4°C overnight with shaking. The membranes were then incubated in secondary antibodies (conjugated goat antirabbit IgG; CWBIO, Cat. No. CW0103S, 1:2,000 dilution) at room temperature for 2 h and washed in TBST three times for 15 min. Then, the membranes were incubated in Super Signal ECL-HRP detection reagent (ComWin Biotech) for 1 min followed by exposure to film in a visualizer.

### Statistical analysis

All statistical analyses were performed in the R language. All statistical tests were bilateral, and P < 0.05 was considered statistically significant.

## Results

### Ectopic expression of BRDs in OSC patients

**Figure 2:**
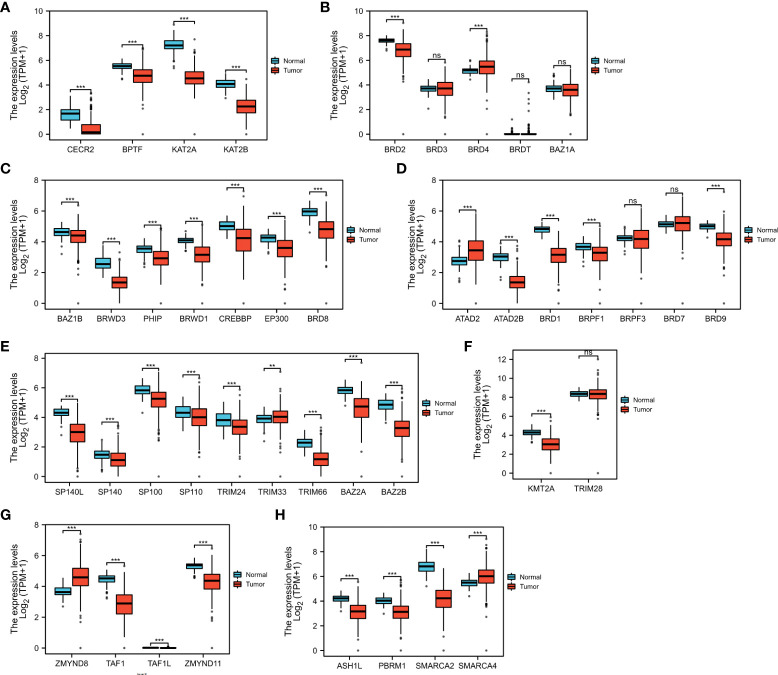
BRD mRNA levels in the OSC. The mRNA levels of BRD subfamilies I **(A)**/II **(B)**/III **(C)**/IV **(D)**/V **(E)**/VI **(F)**/VII **(G)**/VIII **(H)** in OSC samples and normal ovary samples based on TCGA and GETx databases. ns > 0.05; **p < 0.01; ***p < 0.001.

First, we confirmed the expression of these BRDs in OSC tissue samples compared to normal ovary tissue samples based on TCGA and GTEX databases. The results showed that BRDs of subfamily I were decreased in OSC samples (**Figure 2A**). BRD2 was significantly decreased, but BRD4 was significantly increased in OSC samples in BRD subfamily II (**Figure 2B**). Both genes of BRD subfamily III were decreased in OSC samples (**Figure 2C**). The members of BRD subfamily IV, ATAD2B, BRD1, BRPF1, and BRD9, were downregulated, but ATAD2 was increased in OSC tissues (**Figure 2D**). In BRD subfamily V, the expression levels of SP140L, SP140, SP100, SP110, TRIM24, TRIM66, BAZ2A and BAZ2B were significantly reduced, but TRIM33 expression was markedly enhanced (**Figure 2E**). KMT2A, a subfamily VI gene, was significantly decreased in OSC samples (**Figure 2F**). In BRD subfamily VII, TAF1, TAF1L and ZMYND11 were significantly decreased, but ZMYND8 was markedly increased in OSC samples (**Figure 2G**). The members of BRDs of subfamily VIII, ASH1L, PBRM1 and SMARCA2, were significantly decreased, but SMARCA4 was significantly increased in OSC samples compared to normal tissue samples (**Figure 2H**). These results indicated that the ectopic expression of BRDs might be involved in the development and progression of OSC.

### DNA alterations of BRDs in OSCs

**Figure 3:**
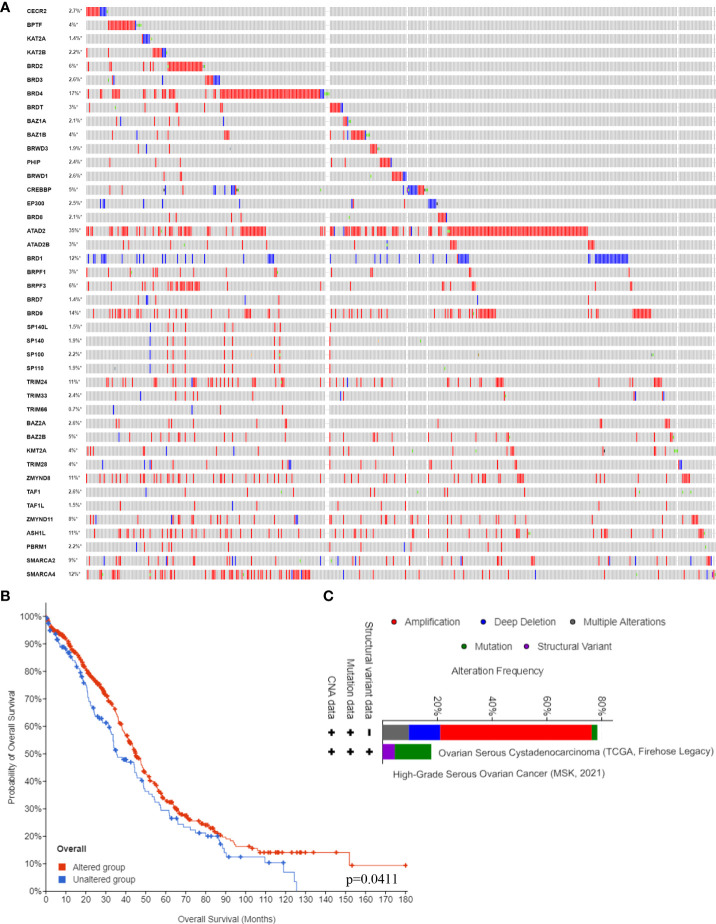
BRD DNA alteration levels in the OSC. **(A)** The DNA alteration levels of BRDs in OSC samples and normal ovary samples based on the cBioPortal database. **(B)** The overall survival analysis of OSC patients with or without BRD DNA alterations. **(C)** The types of BRG alterations in OSC samples based on the cBioPortal database. *p < 0.05.

Next, we confirmed the DNA alterations of these BRDs in OSC samples based on the cBioPortal database. These BRDs all have different levels of DNA mutations in OSC samples, such as CECR2 (2.7%), BPTF (4%), KAT2A (1.4%), KAT2B (2.2%), BRD2 (6%), BRD3 (2.6%), BRD4 (17%), BRDT (3%), BAZ1A (2.1%), BAZ1B (4%), BRWD3 (1.9%), PHIP (2.4%), BRWD1 (2.6%), CREBBP (5%), EP300 (2.5%), BRD8 (2.1%), ATAD2 (35%), ATAD2B (3%), BRD1 (12%), BRPF1 (3%), BRPF3 (6%), BRD7 (1.4%), BRD9 (14%), SP140L (1.5%), SP140 (1.9%), SP100 (2.2%), SP110 (1.9%), TRIM24 (11%), TRIM33 (2.4%), TRIM66 (0.7%), BAZ2A (2.6%), BAZ2B (5%), KMT2A (4%), TRIM28 (4%), ZMYND8 (11%), TAF1 (2.6%), TAF1L (1.5%), ZMYND11 (8.8%), ASH1L (11%), PBRM1 (2.2%), SMARCA2 (9%), and SMARCA4 (12%) (**Figure 3A**). The overall survival analysis showed that the DNA alteration group of these OSC patients had a favorable prognosis compared to the non-DNA alteration group (**Figure 3B**). Moreover, the types of DNA alterations included amplification, deep deletion, multiple alterations, mutation, and structural variants (**Figure 3C**).

### Possible molecular functions of BRDs in OSC

**Figure 4:**
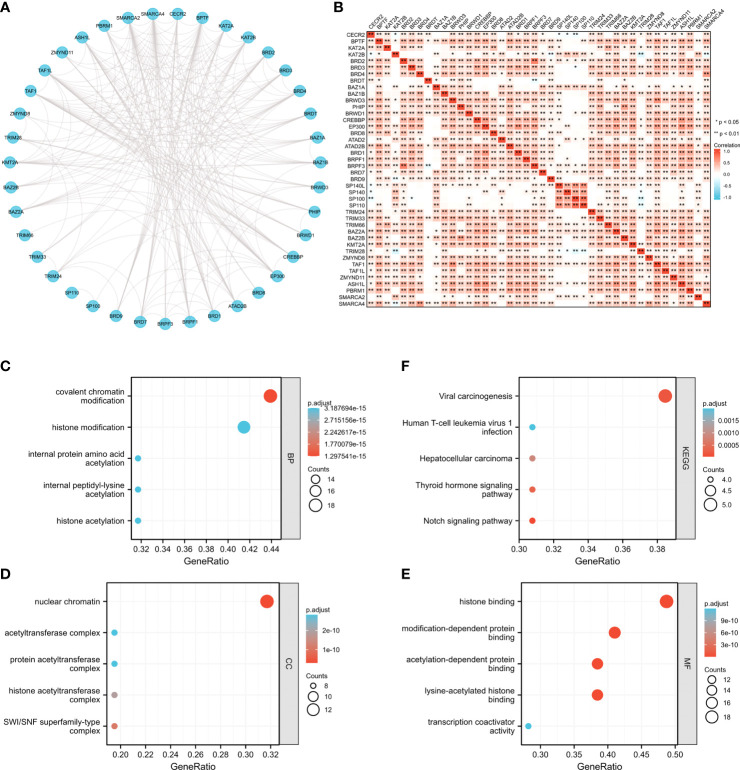
The potential molecular functions of BRDs in OSC. **(A)** PPI network construction for BRDs based on the STRING database. **(B)** Correlation analysis among the 42 BRGs based on the TCGA database OSC datasets. **(C)** GO enrichment of BRG genes for BP terms. **(D)** GO enrichment of BRG genes for CC terms. **(E)** GO enrichment of BRG genes for MF terms. **(F)** KEGG enrichment of BRG genes. *p < 0.05; **p < 0.01.

To further elucidate the possible molecular functions of BRDs in OSC progression, we constructed a PPI network for these BRDs based on the STRING database (**Figure 4A**). Furthermore, we analyzed the correlations among these BRDs in OSC patients based on the TCGA database OSC dataset (**Figure 4B**). We also found that these BRDs were enriched in covalent chromatin modification, histone modification, internal protein amino acid acetylation, internal peptidyl-lysine acetylation, and histone acetylation for biological progression terms (**Figure 4C**); in nuclear chromatin, acetyltransferase complex, protein acetyltransferase complex, histone acetyltransferase complex, and SWI/SNF superfamily type complex for cellular component terms (**Figure 4D**); and in histone binding, modification-dependent protein binding, acetylation-dependent protein binding, lysine-acetylated histone binding, and transcription coactivator activity for molecular function terms (**Figure 4E**). Moreover, KEGG analysis indicated that these genes were also enriched in viral carcinogenesis, human T-cell leukemia virus 1 infection, the thyroid hormone signaling pathway and the Notch signaling pathway (**Figure 4F**). We also confirmed each BRDs functions in OSC progression based on LinkedOmics database, which indicated that these BRDs were mostly involved in metabolic process, growth, chromatin binding, molecular transducer activity, and lipid binding (**Supplemental Figure 1**). These results indicated that these BRDs might collaboratively drive viral carcinogenesis by epigenetic regulation, especially histone acetylation to drive OSC progression..

### Classification of BRD subtypes in OSC

**Figure 5:**
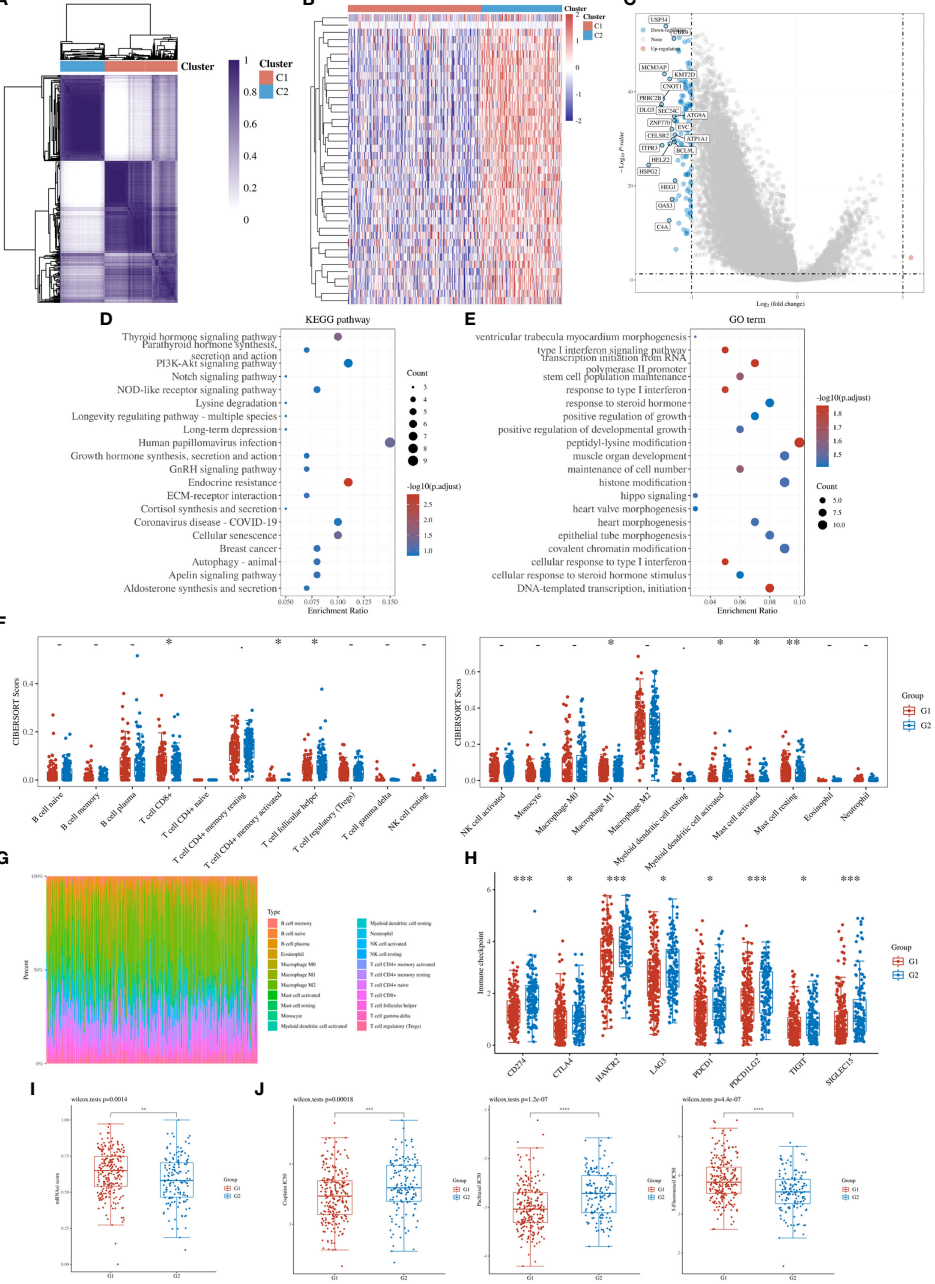
Stratification of OSCs based on the expression of BRDs. **(A)** Heatmap depicting consensus clustering solution for BRDs based on the TCGA database OSC dataset. **(B)** Heatmap of DEGs based on Cluster 1 compared to Cluster 2. **(C)**. Volcano plot of DEGs. **(D)** KEGG enrichment of DEGs. **(E)** GO enrichment of DEGs. **(F)** Immune cell expression distribution for Cluster 1 compared to Cluster 2. **(G)** The percentage abundances of different immune cell types in each sample. **(H)** Heatmap of immune checkpoint-related gene expression for Cluster 1 and Cluster 2. **(I)** The stemness score for Cluster 1 and Cluster 2. **(J)** The drug sensitivity analysis for cisplatin, paclitaxel, and 5-Fu in Cluster 1 and Cluster 2. (G1 is the group of the Cluster 1 for OC patients. G2 is the group of the Cluster 2 for OC patients.) *p < 0.05; **p < 0.01; ***p < 0.001; ****p < 0.0001.

**Figure 6:**
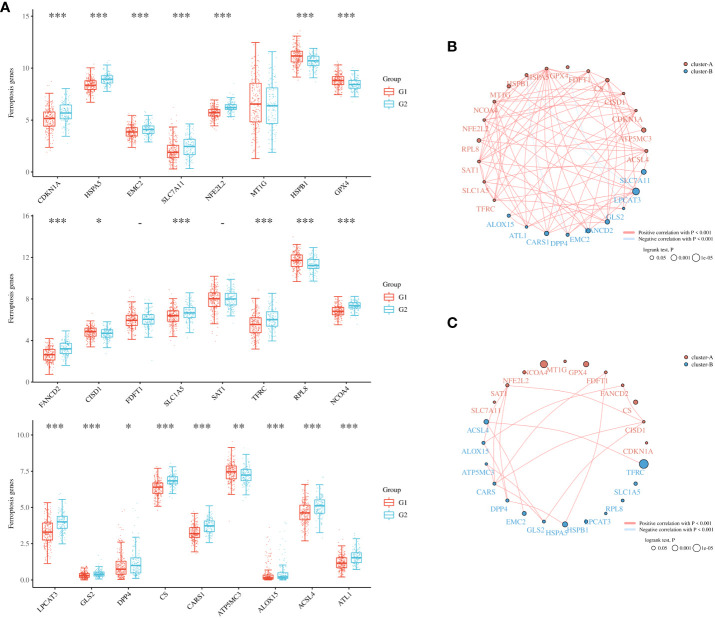
Ferroptosis levels in different subgroups of OSC. **(A)** Heatmap of ferroptosis-related genes in Cluster 1 and Cluster 2. **(B)** Network construction of ferroptosis-related genes in Cluster 1 samples. **(C)** Network construction of ferroptosis-related genes in Cluster 2 samples. (G1 is the group of the Cluster 1 for OC patients. G2 is the group of the Cluster 2 for OC patients.) *p < 0.05; **p < 0.01; ***p < 0.001.

**Figure 7:**
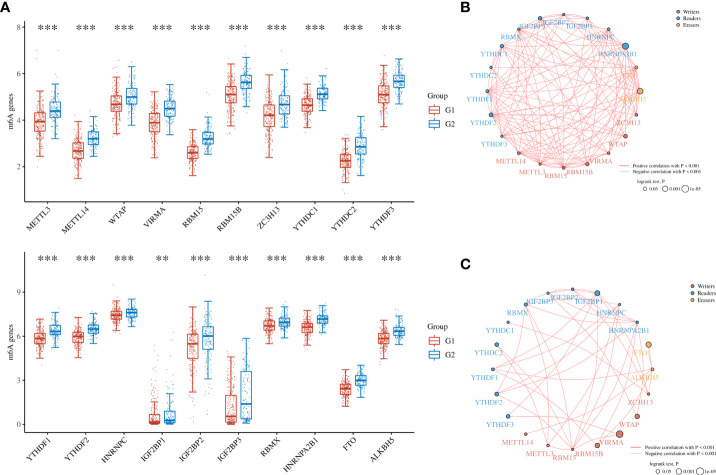
The m6A levels in different subgroups of OSC. **(A)** Heatmap of m6A-related genes in Cluster 1 and Cluster 2. **(B)** The network construction of m6A-related genes in Cluster 1 samples. **(C)** The network construction of m6A-related genes in Cluster 2 samples. (G1 is the group of the Cluster 1 for OC patients. G2 is the group of the Cluster 2 for OC patients.) **p < 0.01; ***p < 0.001.

To further refine the potential role of BRDs in OSC, we used the ConsensusClusterPlus R package to identify OSC patients from the TCGA database into two subtypes based on the 42 BRDs, which classify 234 cases into Cluster 1 and 142 cases into Cluster 2 (**Figure 5A**). Moreover, the 127 differentially expressed genes (DEGs) from Cluster 1 compared to Cluster 2 are shown in **Figures 5B, C**. GO and KEGG enrichment analyses indicated that these DEGs were enriched in the thyroid hormone signalling pathway, parathyroid hormone synthesis, secretion and actin, the PI3K-Akt signalling pathway, the Notch signalling pathway, the NOD-like receptor signalling pathway, and lysine degradation for KEGG terms (**Figure 5D**) and enriched in the type I interferon signalling pathway, transcription initiation from RNA polymerase II promoter, stem cell population maintenance, response to type I interferon, positive regulation of growth, and histone modification (**Figure 5E**), which indicated that the DEGs might play key roles in OSC progression by driving immune infiltration and stemness maintenance. Therefore, we further confirmed the effects of these DEGs on immune infiltration in OSC patients in different clusters. The results showed that the levels of CD8+ T cells, CD4+ memory-activated T cells, M1 macrophages, and resting mast cells were increased in Cluster 1, but T follicular helper cells and activated myeloid dendritic cells were decreased in Cluster 1 compared to Cluster 2 (**Figures 5F, G**). The immune checkpoints were decreased in Cluster 1 compared to Cluster 2, including CD274, CTKA4, HAVCR2, LAG3, PDCD1, PDCD1LG2, TIGIT, and SIGLEC15 (**Figure 5H**). Moreover, stemness analysis indicated that the level of stemness was higher in Cluster 1 than in Cluster 2 (**Figure 5I**). The drug sensitivity analysis showed that the effect of cisplatin and paclitaxel was significantly sensitivity in Cluster 1 than Cluster 2, but the effect of 5-Fu was more sensitivity in Cluster 2 compared to Cluster 1 (**Figure 5J**). We also found differential expression of ferroptosis genes, including CDKN1A, HSPA5, EMC2, SLC7A11, NFE2L2, HSPB1, GPX4, FANCD2, CISD1, SLC1A5, TFRC, RPL8, NCOA4, LPCAT3, GLS2, DPP4, CS, CARS1, ATP5MC3, ALOX15, ACSL4 and ATL1, between Cluster 1 and Cluster 2 (**Figure 6A**). Moreover, we found that the correlation network of these ferroptosis genes in Cluster 1 samples (**Figure 6B**) was markedly different compared to Cluster 2 samples (**Figure 6C**). The expression levels of m6A methylation genes, including METTL3, METTL14, WTAP, VIRMA, RBM15, RBM15B, ZC3H13, YTHDC1, YTHDC2, YTHDF3, YTHDF1, YTHDF2, HNRNPC, IGF2BP1, IGF2BP2, IGF2BP3, RBMX, HNRNPA2B1, FTO and ALKBH5, also showed significant differences in Cluster 1 compared to Cluster 2 (**Figure 7A**). The correlations of these m6A genes were also markedly different in Cluster 1 compared to Cluster 2 (**Figures 7B, C**). These results indicated that BRDs might drive immune infiltration, stemness maintenance, ferroptosis and m6A methylation to regulate the development and progression of OSC.

### Identification of the BRD signature in OSC

**Figure 8:**
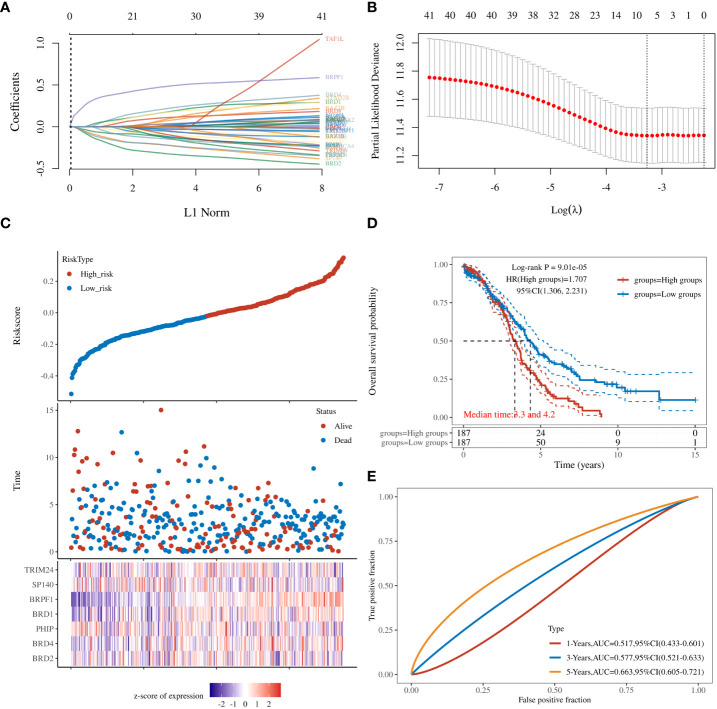
Prognostic signature of OSC patients. **(A)** Prognostic signature construction by LASSO Cox analysis. **(B)** The lambda is represented in the abscissa, and the coefficients are represented in the ordinate. **(C)** The risk score, survival time, and signature expression profiles in OSC patients. **(D)** The overall survival analysis between the high- and low-risk OSC patient groups. **(E)** ROC curves for survival time in OSC patients.

**Figure 9:**
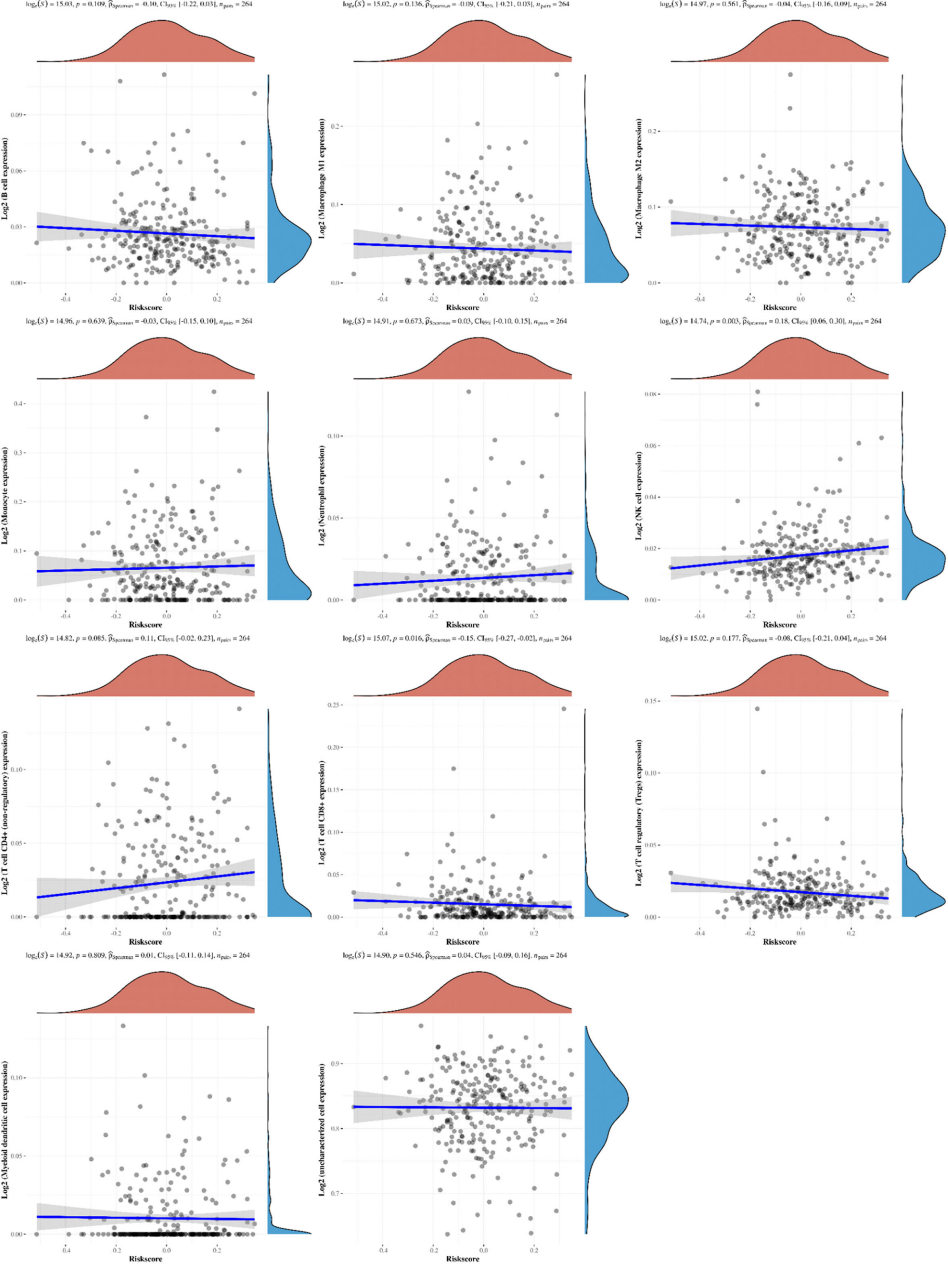
The correlations between the Riskscore and different immune cell infiltrations. The associations among the Riskscore and infiltration of different immune cells, including B cells, M1 macrophages, M2 macrophages, monocytes, neutrophils, NK cells, CD4+ T cells, CD8+ T cells, Treg cells, myeloid dendritic cells, and uncharacterized cells.

**Figure 10:**
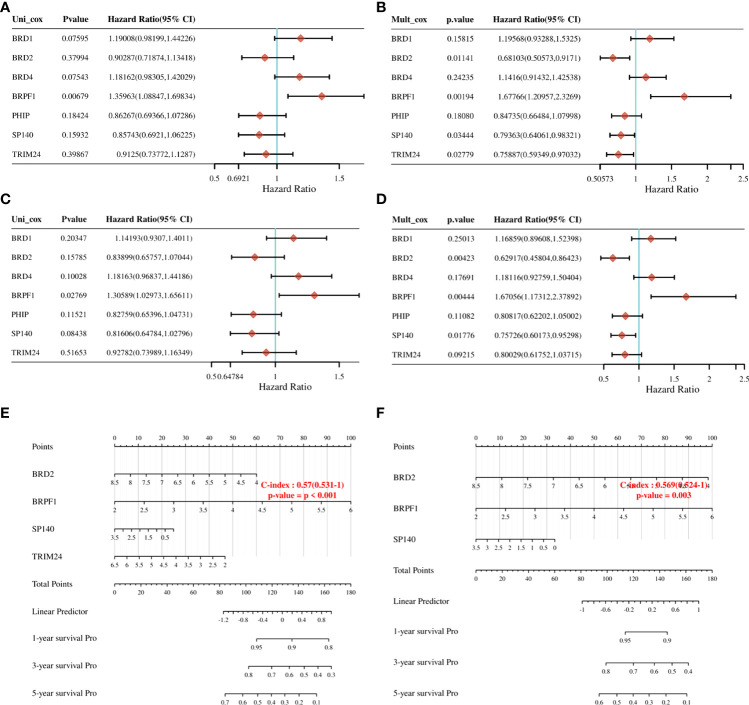
Prognostic value of seven expression signatures in OSC. **(A)** Prognostic values of seven signatures shown by forest plot of hazard ratios by Uni-Cox analysis for OS. **(B)** Prognostic values of seven signatures shown by forest plot of hazard ratios by Multi-Cox analysis for OS. **(C)** Prognostic values of seven signatures shown by forest plot of hazard ratios by Uni-Cox analysis for DSS. **(D)** Prognostic values of seven signatures shown by forest plot of hazard ratios by Multi-Cox analysis for DSS. **(E)** Nomogram survival prediction chart for predicting OS rates at 1, 3, and 5 years. **(F)** Nomogram survival prediction chart for predicting DSS rates at 1, 3, and 5 years.

To further clarify the prognostic significance of BRDs in each OSC patient, we used LASSO regression to construct the risk model (lambda.min=0.0381), and 7 signatures were confirmed. Risk score=(-0.1228)*BRD2+(0.0055)*BRD4+(-0.0525)*PHIP+(0.0277)*BRD1+(0.3124)*BRPF1+(-0.0815)*SP140+(-0.0987)*TRIM24 (**Figure 8A, B**). The risk score, status, and 7 signature expression profiles of the high- and low-risk groups are shown in **Figure 8C**. The overall survival analysis suggested that the OSC patients in the high-risk group had an unfavorable prognosis compared to those in the low-risk group (**Figure 8D**). The AUC values at 1, 3, and 5 years were 0.517, 0.577, and 0.663, respectively (**Figure 8E**). Moreover, we found that the risk score was significantly and negatively correlated with the levels of B cells and CD8+ T cells but positively correlated with NK cells (**Figure 9**). Furthermore, Uni-Cox regression indicated that BRPF1 was a significant risk factor for BRD (**Figure 10A**), while Multi-Cox regression suggested that BRD2, BRPF1, SP140, and TRIM24 were significant BRDs in OSC patient’s OS (**Figure 10B**). Moreover, Uni-Cox regression indicated that BRPF1 was a significant risk factor for BRD (**Figure 10C**), while Multi-Cox regression suggested that BRD2, BRPF1, and SP140 were significant BRDs in OSC patient’s DSS (**Figure 10D**). We further combined the mRNA levels of these BRDs to construct a nomogram to predict the survival probability of patients at 1, 3, and 5 years for OS and DSS. The nomogram suggested that the prognostic prediction of the mRNA level of BRPF1 was better than those of BRD2, SP140, and TRIM24 in OS (**Figure 10E**), and the BRPF1 mRNA level was also better than BRD2 and SP140 in DSS (**Figure 10F**). These results indicated that BRDs had significant prognostic value for OSC patients, especially BRPF1.

### The role of BRPF1 in OSC

**Figure 11:**
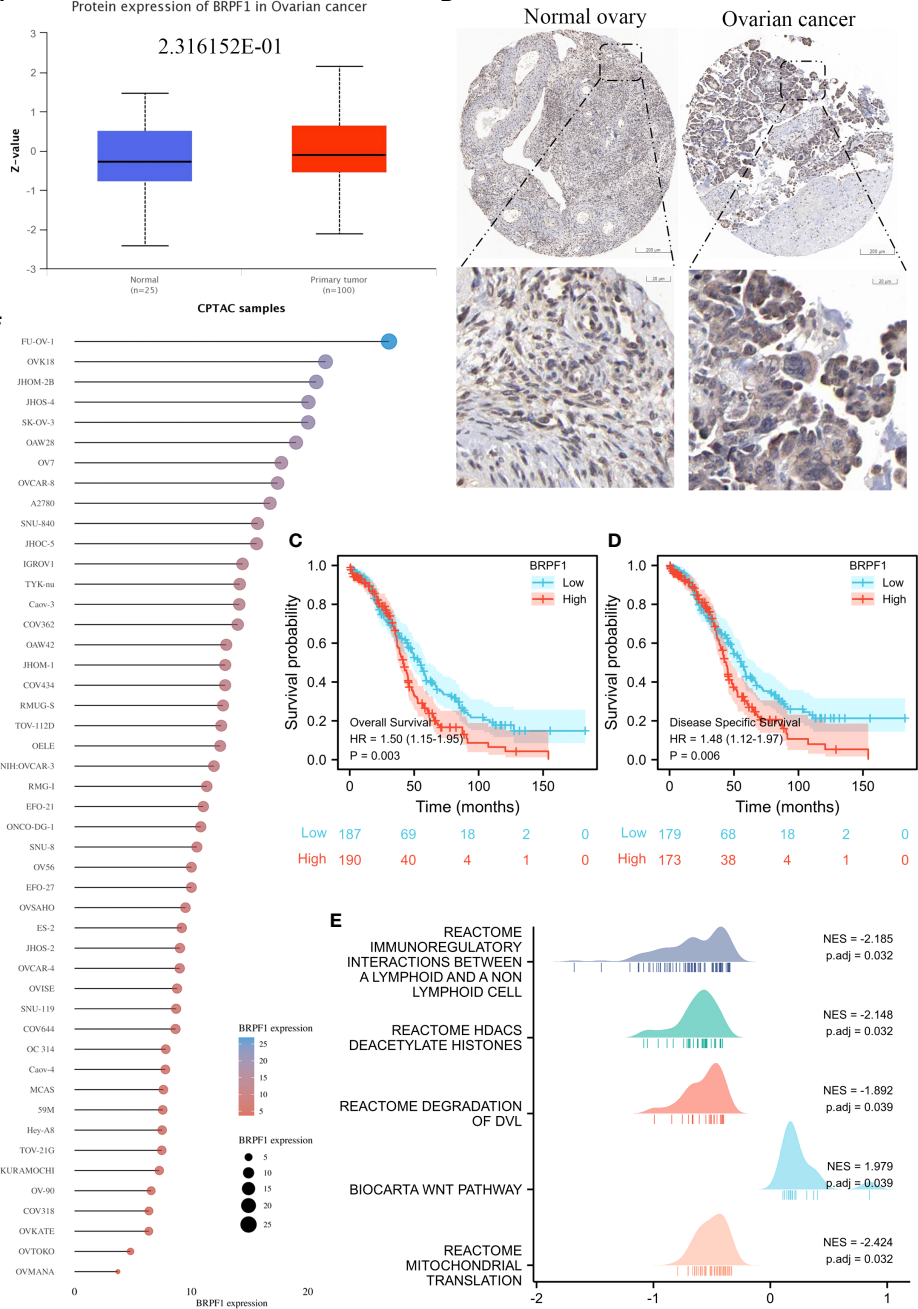
The expression, prognostic value and molecular function prediction of BRPF1 in OSC. **(A)** The total protein expression of BRPF1 in the CPTAC database. **(B)** The protein expression and lOSCation of BRPF1 in the HPA database. **(C)** Overall survival analysis of BRPF1 in OSC. **(D)** The disease-specific survival analysis of BRPF1 in OSC. **(E)** GSEA of BRPF1 in OSC tissue samples. **(F)** The expression of BRPF1 in multiple OSC cell lines based on the CCLE database.

To confirm the role of BRPF1 in OSC progression, we used the CPTAC database to confirm BRPF1 protein expression in OSC patients, which indicated that the protein expression of BRPF1 was not significantly changed in OSC samples and normal ovary samples (**Figure 11A**). We further confirmed the cellular localization of BRPF1 in OSC samples based on the HPA database, which showed that BRPF1 was located in the cell nucleus in normal ovary samples but located in the nucleus and cytoplasm in OSC samples (**Figure 11B**). These results indicated that cytosolic translocation of BRPF1 might be an important step in carcinogenesis. Moreover, overall survival and disease-specific survival analyses indicated that a high level of BRPF1 could induce a poor prognosis in OSC patients (**Figures 11C, D**). The GSEA indicated that BRPF1 might have multiple molecular functions, such as immunoregulatory interactions between lymphoid and nonlymphoid cells, HDAC deacetylation of histones, degradation of DVL, the Wnt pathway, and mitochondrial translation (**Figure 11E**). To further verify the molecular functions of BRPF1 in OSC cells, we used the CCLE database to confirm the expression of BRPF1 in OSC cell lines, and we chose SKOV3 to carry out the experiment (**Figure 11F**).

**Figure 12:**
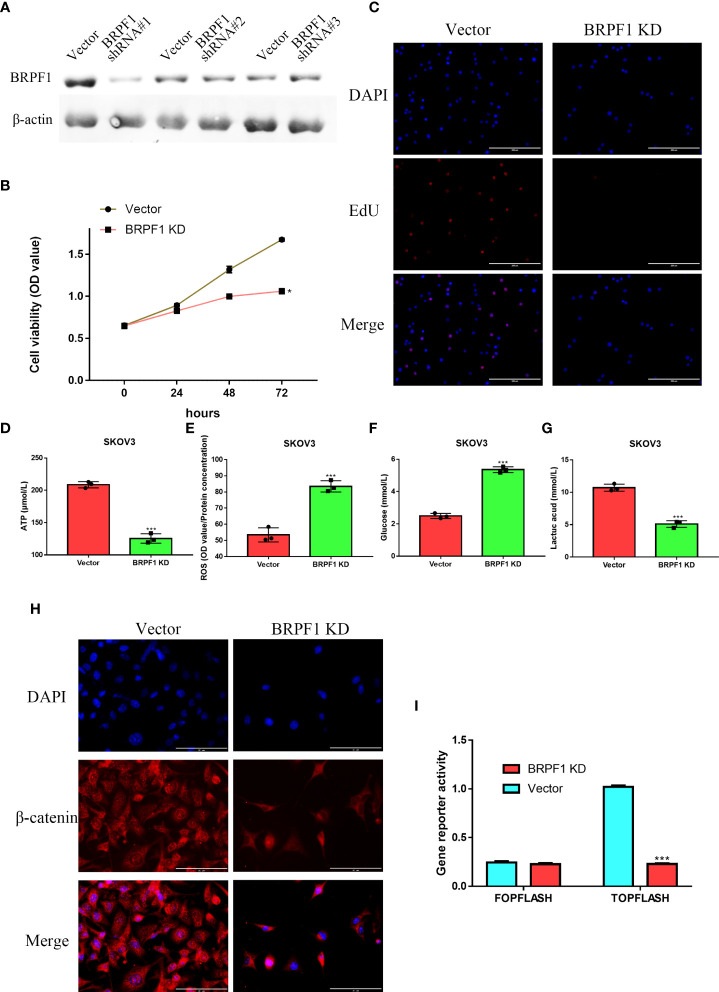
The effect of BRPF1 inhibition in SKOV3 cell lines**(A)** The effects of BRPF1 shRNA#1/2/3 on SKOV3 cell lines. **(B)** The effect of BRPF1 knockdown on the viability of SKOV3 cells by MTT assay. **(C)** The effect of BRPF1 inhibition on SKOV3 cell proliferation by EdU analysis. **(D)** The effect of BRPF1 inhibition on SKOV3 cell ATP production. **(E)** The effect of BRPF1 inhibition on SKOV3 cell ROS production. **(F)** The effect of BRPF1 inhibition on SKOV3 cell glucose consumption. **(G)** The effect of BRPF1 inhibition on SKOV3 cell lactic acid production. **(H)** The effect of BRPF1 knockdown on the lOSCation of β-catenin. **(I)** The effect of BRPF1 knockdown on Wnt pathway activity by a dual-luciferase reporter. *p < 0.05; ***p < 0.001.

SKOV3 cells were transfected with three BRPF1 shRNAs, and western blotting indicated that BRPF1 shRNA#1 had the strongest inhibitory effect on BRPF1 expression in SKOV3 cells (**Figure 12A**). MTT analysis indicated that BRPF1 inhibition could suppress the viability of OSC cells (**Figure 12B**). EdU analysis also showed that BRPF1 knockdown reduced the proliferation ability of OSC cells (**Figure 12C**). Based on the GSEA results, we further examined the effect of BRPF1 on cell metabolism (**Figure 11E**). The results indicated that BRPF1 knockdown decreased the consumption of glucose and the production of ATP and lactic acid but increased ROS production in OSC cells (**Figures 12D-G**). We also detected the effect of BRPF1 inhibition on the Wnt pathway, which indicated that BRPF1 knockdown could inhibit β-catenin nuclear translocation and Wnt pathway activity (**Figure 12H, I**). These results indicated that BRPF1 could promote cell proliferation, anaerobic metabolism and the Wnt pathway.

**Figure 13:**
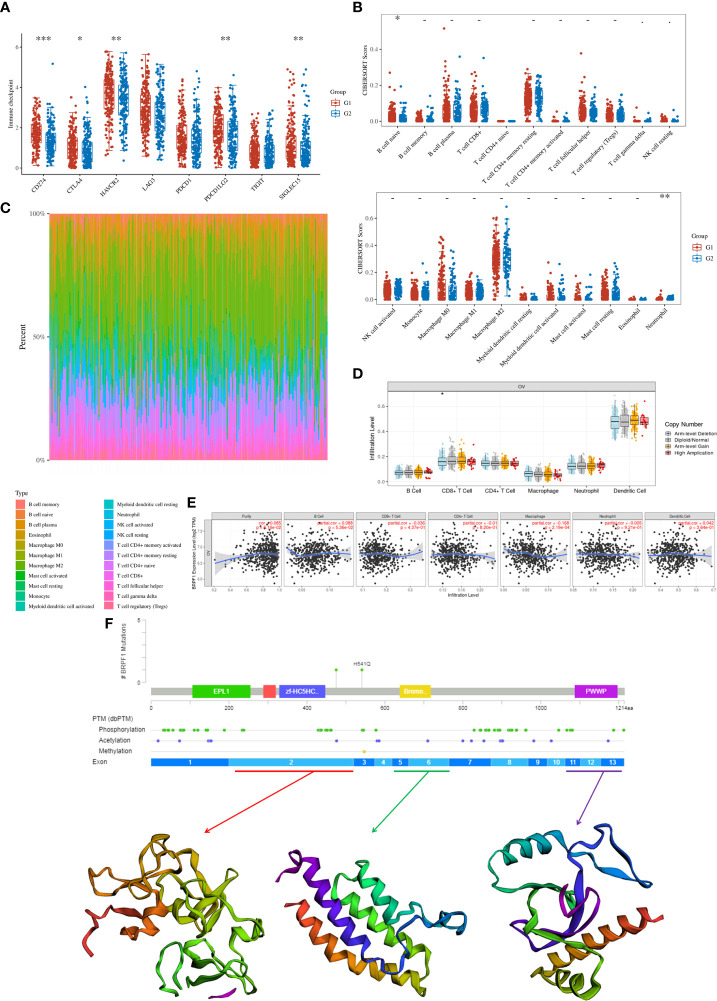
The role of BRPF1 in OC immune infiltration. **(A)** Immune cell expression distribution for the OC patients with high expression of BRPF1 compared to the OC patients with low expression of BRPF1. **(B)** The percentage abundances of different immune cell types in each sample. **(C)** Heatmap of immune checkpoint-related gene expression for the OC patients with high expression of BRPF1 compared to the OC patients with low expression of BRPF1. **(D)** The CNV analysis between BRPF1 alteration and immune infiltration based on TIMER database. **(E)** The expression level of BRPF1 and immune infiltration based on TIMER database. **(F)** The protein secondary structure of BRPF1 and the tertiary structure of BRPF1 protein for exon 1, exons 5-6, and exons 11-13. (G1 is the group of the OC patients with high expression of BRPF1. G2 is the group of the OC patients with low expression of BRPF1.) *p < 0.05; **p < 0.01; ***p < 0.001.

Finally, we confirmed the molecular functions of BRPF1 in immune infiltration, which indicated that high expression of BRPF1 was negatively and significantly correlated with the expression of some immune checkpoints, including CD274, CTLA4, HAVCR2, PDCD1LG2 and SIGLEC15, in OSC patients (**Figure 13A**). Moreover, BRPF1 was positively correlated with naive B cells but negatively correlated with neutrophils (**Figures 13B, C**). The CNV analysis indicated that the CD8+ T cell infiltration level was significantly decreased in OSC patients with arm-level deletion of BRPF1 (**Figure 13D**). The expression of BRPF1 significantly and negatively correlated with macrophage (**Figure 13E**). Moreover, the protein structure analysis indicated that BRPF1 had multiple function domains, such as EPL1, PHD, zf-HC5HC2H, Bromodomain and PWWP domain. The BRPF1 protein modification include phosphorylation, acetylation and methylation, which may regulate the activity of BRPF1 (**Figure 13F**). Taken together, BRPF1 might influence cancer immune infiltration to regulate OSC development and progression.

## Discussion

In this study, we confirmed the mRNA levels of 42 BRDs in OSC patient samples based on TCGA database. These BRDs were differently dysregulated in OSC tissues. Nowadays, BRDs are regarded as a kind of emerging clinical therapeutic targets, and inhibiting any of BRDs activation or expression can be a small molecule inhibitor as emerging epigenetic therapies for cancer (65). Ectopic expression of these BRDs has been confirmed in different cancer types, including bladder cancer (66), diffuse large B-cell lymphoma (67), colorectal cancer (68), liver cancer (50), gastric cancer (69), cervical cancer (70), and breast cancer (71). We further confirmed the DNA alteration levels of these BRDs in OSC patients based on the cBioPortal database, indicating that BRDs were frequently mutated, especially in amplification in OSC patients. A previous study also found that multiple BRDs were altered in different cancer types, such as cervical cancer (72), esophageal squamous cell carcinoma (73), triple-negative breast cancer (74), and diffuse large B-cell lymphoma (75). These results suggested that dysregulation and alteration of BRDs were involved in the occurrence, development and progression of OSC.

To identify the potential functions of BRDs in OSC progression, we found that these BRDs might synergistically exert pathophysiological effects by PPI network construction and correlation analysis in OSC progression, mediating multiple molecular functions, especially histone acetylation, covalent chromatin modification, and viral carcinogenesis. Takao Fujisawa and his colleagues indicated that BRDs selectively recognize and bind to acetylated histone Lys residues to epigenetically regulate gene transcription, and these BRDs are frequently and obviously dysregulated in cancer progression (4). Zaware N et al. found that BRDs could regulate chromatin-templated gene transcription, DNA replication, repair and recombination by driving protein−protein interactions and promoting carcinogenesis (65). Cai et al. found that BRD1 inhibition could attenuate the function of sulfatide to reduce H3K9/14 acetylation and repress the occupancy of histone acetyltransferase binding to ORC1 (HBO1) and monocytic leukemia zinc finger (MOZ) in the promoter of the integrin αV gene in liver cancer cells, reducing migration and invasion (76). Therefore, these BRDs might synergistically promote OSC carcinogenesis to drive histone acetylation.

To further confirm the role of BRDs in OSC progression, we divided OSC patient samples into two subtypes based on these 42 BRDs. We found that the DEGs in Cluster 1 compared to Cluster 2 were enriched in response to type I interferon, peptidyl lysine modification, histone modification, cellular response to type I interferon and multiple signaling pathways. Moreover, we found that there were significant differences in immune infiltration, stemness maintenance, ferroptosis scores, and m6A levels between the two subgroups. Previous studies have indicated that ferroptosis plays a significant role in regulating immune infiltration in cancer progression (77, 78). Zhao et al. found that NCOA4 could mediate ferroptosis, relying on the coordination of BRD4 and CDK9 (79). Chen et al. also found that BRD4/8/9 were significantly correlated with immune infiltration in hepatocellular carcinoma (80). Zhu and his colleagues found that EP300 mutation could induce antitumor immunity and upregulate TMB in bladder cancer (66). Taken together, these results indicated that BRDs might regulate immune infiltration by driving ferroptosis. Furthermore, Patrycja et al. found a significant correlation between distinct BRDs and stemness maintenance in 27 solid cancer types (81). Hao and his colleagues found that EP300 could induce an increase in ALKBH5 expression to reduce the m6A level of FOXM1 mRNA by upregulating H3K27ac, resulting in enhanced FOXM1 mRNA levels and EMT cascade activation in melanoma (82). Zeng et al. found that EP300 could epigenetically upregulate RBM15 to accelerate clear cell renal cell carcinoma growth, metastasis and macrophage infiltration by driving CXCL11 mRNA m6A modification (83). These results indicated that BRDs might maintain cancer stemness by directly driving histone acetylation and indirectly regulating m6A methylation.

Next, we explored the prognostic value of these BRDs in OSC patients. We constructed a prognostic model by LASSO regression based on 7 BRD signatures (BRD2, BRD4, PHIP, BRD1, BRPF1, SP140, and TRIM24), which divided OSC patients into high- and low-risk groups to identify the BRD-based prognostic signature. Moreover, the risk score was significantly and negatively correlated with the levels of B cells and CD8+ T cells but positively correlated with NK cells, which indicated that the effect of BRDs on OSC prognosis might be attributed to the influence of immune infiltration, especially in B cells, CD8+ T cells, and NK cells. In a previous study, BRD4 was identified as a biomarker for predicting poor prognosis for prostate cancer patients (84, 85). BRD1 was significantly correlated with an unfavorable prognosis in colorectal cancer patients (68). PHIP is regarded as an important biomarker for cutaneous melanoma (86) and breast cancer (87). BRPF1 in urine is considered a potential marker of prostate cancer (88). SP140 might be a biomarker in head and neck squamous cell carcinoma (89). TRIM24 is a possible prognostic marker for prostate cancer (90), head and neck squamous cell carcinomas (91) and breast cancer (92). However, whether these indicators can be used as prognostic markers independently for OSC is still unclear. We further confirmed that BRPF1 had a better prognostic significance than the other 6 signatures by Uni-Cox or Multi-Cox analysis. Our further results suggest that BRPF1 has obvious cytosolic aggregation in OSC tissue samples, which indicated that it might bind directly to cytosolic proteins. GSEA also indicated that BRPF1 might drive Wnt pathway activation, mitochondrial translation and immune infiltration in OSCC. Moreover, we found that BRPF1 knockdown inhibited OSC cell proliferation, cell glycometabolism, and Wnt signalling activation. Shima H and his colleagues found that BRPF1 interacts with MOZ to activate the HOX pathway and promote the progression of acute myeloid leukemia (52). Cheng et al. found that BRPF1 interacts with the MOZ/MORF complex to acetylate H3K14, resulting in E2F2 and EZH2 activation and upregulation to promote liver cancer progression (50). Alberto-Aguilar DR et al. found that the ascites of OSC patients could modulate the fucosylation of BRPF1 to promote OSC development (93). These results suggested that BRPF1, as a highly conserved oncogene, might play equally important oncogenic roles in different cancers. Taken together, these results suggest that BRPF1 inhibition suppresses the nuclear translocation of β-catenin and further inactivates the Wnt pathway in OSC. Moreover, BRPF1 had a significant correlation with immune infiltration, especially in naïve B cells and neutrophils, which indicated that BRPF1 not only promoted metabolic reprogramming and proliferation of OSCs in an epigenetic manner but also affected the progression of OSCs by affecting immune infiltration.

## Conclusion

In this study, we confirmed the expression, function, and prognostic significance of 42 BRDs in OSC. Subtype classification also indicated the effects of these BRDs on OSC cell immune infiltration, stemness maintenance, ferroptosis and m6A methylation. Moreover, we found that BRPF1 knockdown could inhibit OSC cell proliferation, glycometabolism, and Wnt pathway activation, indicating that BRPF1 might be a potential therapeutic target and prognostic marker for OSC patients.

## Data availability statement

The original contributions presented in the study are included in the article/**Supplementary Material**. Further inquiries can be directed to the corresponding authors.

## Author contributions

JZ, and YL analyzed the data. T-yF used online tools. DL and W-dZ designed the project. JZ wrote the paper. HL and Y-kL revised the manuscript and designed the experiment. All authors contributed to the article and approved the submitted version.

## Funding

The present study was supported by the Natural Science Foundation of HuNan Province (2021JJ50070).

## Conflict of interest

The authors declare that the research was conducted in the absence of any commercial or financial relationships that could be construed as a potential conflict of interest.

## Publisher’s note

All claims expressed in this article are solely those of the authors and do not necessarily represent those of their affiliated organizations, or those of the publisher, the editors and the reviewers. Any product that may be evaluated in this article, or claim that may be made by its manufacturer, is not guaranteed or endorsed by the publisher.

## Supplementary material

Supplementary Figure 1 The gene functional enrichment analysis for each BRDs based on LinkedOmics database.
